# The Impact of Compensation Gap on Corporate Innovation: Evidence from China’s Pharmaceutical Industry

**DOI:** 10.3390/ijerph19031756

**Published:** 2022-02-03

**Authors:** Liping Fu, Shan Zhang, Fan Wu

**Affiliations:** 1 Department of Business Administration, College of Management and Economics, Tianjin University, Tianjin 300072, China; lpf3688@126.com; 2 Department of Public Administration, College of Management and Economics, Tianjin University, Tianjin 300072, China; wufan92@tju.edu.cn

**Keywords:** pharmaceutical industry, compensation gap, corporate innovation, marketization level, corporate scale

## Abstract

The pharmaceutical industry is typically driven by innovation, and is relevant to people’s livelihoods. How to effectively motivate pharmaceutical enterprises to engage in innovative activities is a hot topic. On the basis of the perspective of the combined effect of tournament theory and social comparison theory, this study explored the impact of internal compensation gap on corporate innovation by using data from China’s listed pharmaceutical enterprises during 2011–2018. The findings show a nonlinear (inverted-U-shaped) relationship between compensation gap and corporate innovation within the pharmaceutical industry, which illustrates that the role of the compensation gap is not endless. We also find the optimal compensation gap between executives and employees. Further analyses indicate that this association is more pronounced in regions with low marketization levels, and in large enterprises. Moreover, the practical significance of the results is explored with an expectation of providing theoretical references for the pharmaceutical industry to establish reasonable incentive mechanisms and promote innovative development, and for the government to introduce innovation incentive policies.

## 1. Introduction

The pharmaceutical industry is a high-tech industry that integrates advanced multidisciplinary technologies and methods, with the significant feature of obtaining high profits on the basis of a patent or proprietary technology. It is typically driven by innovation, and is relevant to people’s wellbeing. Its innovative development is not only conducive to maintaining the competitive advantages of pharmaceutical enterprises, and enhancing their own values, but also necessary for improving people’s living quality [1]. A handful of developed countries remain the source of innovation in the global pharmaceutical industry [2]. Although the pharmaceutical industry in developed countries generally invests highly in *R*&*D*, receives substantial government funding, and produces numerous patents due to the formidable national innovation system [3,4], it is being afflicted by a productivity crisis [5]. Compared to developed countries, the pharmaceutical industry in developing countries is growing rapidly, with strong development dynamics [2,6]. However, it still faces many problems, such as low levels of pharmaceutical innovation, incomplete innovation management systems, and insufficient high-end technologies and talents [7]. Therefore, how to establish an efficient innovation incentive mechanism for pharmaceutical enterprises, especially in emerging countries, is an increasingly hot topic in academic circles.

Corporate innovation is distinctly characterized by high risk and long durations [8]. It is vulnerable to factors such as venture capital [9] and executive short-sightedness [10]. There are many in-depth studies on how to motivate enterprises to innovate. For instance, some studies demonstrated that compensation incentives for non-CEO executives could help improve corporate innovation efficiency [11,12]. Chang et al. [13] found that stock ownership incentives for employees were conducive to improving corporate innovation output. However, incentives for executives and ordinary employees are currently separated in general. Few studies focused on the integrated consideration of the influence of incentives for executives and employees on corporate innovation.

In modern enterprises with separate ownership and right of control, the decision-maker of *R*&*D* activities is at the management level [14]. Hence, managerial incentives are key in enterprise decision-making, as they can mitigate the short-sightedness of managers, and promote managers’ participation in more innovation activities [15]. Meanwhile, ordinary employees play important roles in innovation activities. On the one hand, many patentable ideas are initially generated by frontline employees; on the other hand, ordinary employees often serve as technical support personnel, which may affect innovation productivity [16]. To maximize the values of executives and employees, enterprises need to establish an applicable incentive mechanism to stimulate their enthusiasm in innovation [17]. The executive–employee compensation gap was constructed in this study to examine how the internal compensation gap affects corporate innovation.

Most previous studies on the compensation gap focused on exploring the relationship between that and corporate performance [18,19,20,21,22]. However, the relationship between the compensation gap and corporate innovation has been poorly studied, and no consistent conclusions have been reached. Xu et al. [23], and Zhao and Wang [24] argued that a compensation gap could promote corporate innovation, whereas Siegel and Hambrick [18], and Hon and Lu [25] demonstrated that there is a negative relationship between compensation gap and corporate innovation. The few studies that have been conducted explored the linear relationship between compensation gap and corporate innovation from a single theoretical perspective of tournament theory or social comparison theory. In this paper, a nonlinear relationship between compensation gap and corporate innovation is studied on the basis of the combined perspectives of tournament theory and social comparison theory.

China’s pharmaceutical industry provides appropriate samples for this research due to the following reasons. First, China is populous and has the world’s second largest pharmaceutical market after America [26]. The huge market demand makes the pharmaceutical industry have a vast potential for future development. Second, the Chinese government attaches great importance to innovation in the pharmaceutical industry, and provides a good policy environment for the development of this industry [27], such as the establishment of pharmaceutical parks to encourage innovation, and various tax relief policies for innovative pharmaceutical enterprises. As the Chinese government has a strong intervention role for domestic enterprises, the Chinese pharmaceutical industry will maintain a high growth rate with the vigorous support of government. Third, although China’s pharmaceutical industry is undergoing a rapid increase in output value, it also faces the problems faced by the industry in other emerging countries [28], and thus, the Chinese sample is representative.

On the basis of data of listed pharmaceutical enterprises in China from 2011 to 2018, this paper empirically examines the nonlinear relationship between the executive–employee compensation gap and corporate innovation. Results reveal that there is a nonlinear inverted-U-shaped relationship between internal compensation gap and innovation; the UTEST test result demonstrates that the optimal compensation gap is obtained when the average compensation of executives is 27.103 times that of ordinary employees; the relationship becomes more significant when the enterprise is large-scale or in a less marketized region. These conclusions are still valid after being subjected to robustness tests on replacing the measurement of the key variable and the regression of the lagging dynamic panel model.

The following shows the possible contributions of this research. First, we examine the incentives for both executives and employees to discuss the impact of the internal compensation gap on corporate innovation, which supplements studies on the influencing factors of innovation. Second, this research focuses on the pharmaceutical industry, and, for the first time, discovers an inverted-U-shaped relationship between the internal compensation gap and innovation within it. Our results indicate that neither tournament theory nor equity theory can separately explain this relation. Third, on the basis of the heterogeneity of marketization levels and corporate scales, this research explores the relationship between the internal compensation gap and innovation of pharmaceutical enterprises with different scales and marketization levels, enriching the research perspective of this field. Fourth, the result of this research provides empirical evidence supporting decision-making references for pharmaceutical enterprises and policy-makers to optimize the innovation incentive mechanism.

The remainder of this paper is organized as follows. In Section 2, we present a review of previous studies, and develop our hypothesis. In Section 3, we describe the research design, and outline the data. In Section 4, we analyze the regression results, present the robustness checks, and provide additional tests. Lastly, in Section 5 we discuss our findings, and propose implications and limitations.

## 2. Literature Review

### 2.1. Corporate Innovation

Innovation is a key driving force of national economic growth [29], and an important way to enhance a company’s competitive advantage [30]. It not only determines the technological competitiveness and market position of an enterprise, but also contributes to the quality and sustainability of enterprise development [31]. The core component of corporate innovation is the process through which individuals integrate their human capital and physical resources within the company [32]. The pharmaceutical industry can obtain high profits on the basis of patents or proprietary technologies, and is typically driven by innovation. Product research and technological innovation are the driving force behind the continuous development of this industry [1]. Corporate technological innovation is a complex process with high risk and a long duration [8]. Therefore, how to effectively stimulate innovation is a critical issue for the pharmaceutical industry.

There are a number of factors that could affect managers’ or employees’ participation in innovation activities, and some were intensively studied. These factors include political compromise [33], industrial policy [34], market competition [35], analyst concerns [15], venture capital [9], institutional ownership [36], managers’ overconfidence [9], nonexecutive employee stock options [13], and top executive incentives [37]. Either management groups or ordinary employees play important roles in innovation activities [23]. Firms need to establish proper incentive mechanisms to motivate both managers and employees to engage in innovation activities [17]. However, few empirical studies integrated managerial and employee incentives together in the innovation process.

### 2.2. Compensation Gap

Although the compensation gap between executives and employees has attracted considerable attention, our knowledge of its possible consequences from a corporate economic performance perspective is still rather limited and elusive [38]. On the one hand, Lazear and Rosen [39] proposed tournament theory, and indicated that an enterprise can establish a hierarchy to motivate employees. Rosen [40] extended the discussion of rewards that are used to maintain performance incentives in a multistage hierarchy. According to this theory, the compensation gap is helpful in stimulating a competitor’s effort level, and reducing allocation cost; the larger the compensation gap between adjacent tiers is, the more strongly the employees are stimulated, and the better the enterprise performs. The existence of a tournament model has been proven by a large number of researchers by verifying the positive correlation between compensation gap and corporate performance [19,21,22].

On the other hand, social comparison theory is widely used to explain an individual’s response to compensation. It is dominated by relative disparity theory and equity theory. Equity theory holds that the inequity degree of personal income relative to another person’s income impacts personal emotion and behavior [41], no matter if the inequity is beneficial to oneself [42] or others [43]. Relative disparity theory indicates that an individual may hold a grudge and feel deprived when the individual could obtain a benefit which is actually obtained by others [44]. In these theories, an individual expects the return to be equal to their contribution, and compares it with others to evaluate the equity of the exchange relation between individual and enterprise. A negative influence may be posed if the input–output ratio is lower than that of others. Previous studies showed that the compensation gap can lead to a brain drain [45], reducing internal teamwork efficiency [46] and corporate performance [18].

### 2.3. Compensation Gap and Corporate Innovation

There is a limited number of studies on the impact of compensation gap on corporate innovation, and no consensus has been reached. Some studies found that the compensation gap can promote innovation [23,24], whereas others concluded the opposite [18,25]. In this study, we propose a more nuanced perspective to reconcile this conflict. Tournament theory and social comparison theory are complementary. When establishing a compensation structure, the complementarity and balance of the two theories is more important.

With the increase in the compensation gap, corporate performance presents an inverted-U-shaped relationship that first rises and then declines, which is the combined effect of tournament theory and the social comparison theory [20]. There is no empirical research on the relationship between the inverted-U-shaped executive–employee compensation gap and corporate innovation, let alone studies specific to the pharmaceutical industry. For the first time, this paper explores the influence of an internal compensation gap on corporate innovation, taking the pharmaceutical industry as an example. On the one hand, according to tournament theory, a large compensation gap means a strong attractiveness of entering the management level, motivating employees more to work [47], thus increasing incentives for technological innovation; when executives receive salary satisfaction, it is conducive to stimulating their entrepreneurial spirit, boosting their enthusiasm for innovation, and increasing their level of risk-taking for innovation failure [48]. On the other hand, in accordance with social comparison theory, an excessive compensation gap tends to breed discontent among employees; especially when it is difficult for ordinary employees to obtain executive positions and compensation, employees may be negative and passive towards research and development [46,49,50]. For executives, generous compensation may lead to cognitive biases and self-serving motives, which may further reduce their willingness to innovate [51,52].

In summary, there should be an optimal compensation gap between executives and employees that has the most positive effect on corporate innovation. When the gap is lower than the optimal level, tournament theory plays a dominant role, whereas when the gap is higher than the optimal level, social comparison theory plays a leading role. On the basis of the above analysis, a nonlinear (inverted-U-shaped) relationship between compensation gap and innovation is predicted, and this relationship is the combined effect of the two theories. Hence, the following hypothesis is put forward:

**Hypothesis 1.**  *The effect of the compensation gap on corporate innovation is nonlinear (inverted U).*

## 3. Research Methodology

### 3.1. Sample Selection and Data Source

On the basis of the industry classification criteria of the China Securities Regulatory Commission, we chose listed A-share pharmaceutical companies in China as samples to test our hypothesis. The period of the study was from 2011 to 2018 due to the serious lack of data on *R*&*D* investment of listed pharmaceutical companies prior to 2011. A total of 260 pharmaceutical companies were obtained as our initial samples. Companies with states of ST, *ST, or PT, and companies with missing data were removed. Applying the above criteria, 190 pharmaceutical companies were selected, which yielded balanced panel data for 1520 firm-year observations. Research data were sourced from the China Stock Market and Accounting Research Database (CSMAR). To reduce the impact of extreme values on the research result, all continuous variables were winsorized by 1% and 99%.

### 3.2. Model

The following model was established to test the hypothesis, where *α_i_* is the regression coefficient, and *ε* is the error term. The dependent variable (*R*&*D*) measures corporate innovation. *Gap* is the proxy for compensation gap, and *Gap*^2^ is the square item of compensation gap. *Control* denotes a series of corporate characteristic variables that are the influencing factors of corporate innovation. Moreover, the dummy variables of years and provinces were added to the model. The hierarchical multiple regression was applied to verify the nonlinear relationship between compensation gap and innovation of pharmaceutical enterprises.

(1) $$R\&D=\alpha_{0}+\alpha_{1}Gap+\alpha_{2}Gap^{2}+\sum \alpha_{k}Control+Year+Province+\varepsilon$$

### 3.3. Variables

#### 3.3.1. Dependent Variables

The dependent variable in this study was corporate innovation. *R*&*D* investment is the basic expenditure for innovative activities, including various capital investments in creating new processes and new products. Therefore, *R*&*D* investment was used to measure corporate innovation in this study. In order to make the data comparable across enterprises, the *R*&*D* investment was divided by total revenues.

#### 3.3.2. Independent Variables

The main independent variable in this study was the internal compensation gap. With reference to existing studies [22,47], the average compensation of executives was divided by the average compensation of employees to measure the internal compensation gap. The calculation equation is as follows:

(2) $$Gap=\frac{\frac{{\mathrm{Executives}}^{\prime }\;\mathrm{compensation}}{\mathrm{Total}\;\mathrm{number}\;\mathrm{of}\;\mathrm{executives}}}{\frac{{\mathrm{Employees}}^{\prime }\;\mathrm{compensation}}{\mathrm{Total}\;\mathrm{number}\;\mathrm{of}\;\mathrm{employees}}}$$

#### 3.3.3. Control Variables

With reference to the existing literature [15,53], we controlled for a vector of firm characteristics that have been shown to affect innovation activities. The control variables include institutional investor’s shareholding ratio, firm age, total assets, ROA, leverage, cash ratio, turnover of total assets, and growth rate of sales. To eliminate the influence of annual differences and provincial differences on innovation, the dummy variables of years and provinces were also added in the study. The definitions of the main variables are shown in Table 1.

## 4. Results

### 4.1. Descriptive Statistics

Panel A in Table 2 reports the descriptive statistical results of the main variables. As shown, the average proportion of the sample enterprises’ *R*&*D* investment in their total revenues is 4.28%, with a standard deviation of 2.874, which indicates that the intensity of *R*&*D* investment in pharmaceutical companies is generally not high. The average internal compensation gap of the sample enterprises is 10.49, with a standard deviation of 7.622, which shows that the compensation of executives is about 10 times that of employees, and the internal compensation gap varies considerably between enterprises. Panel B in Table 2 presents the correlation analysis results of the main variables. There was no significant correlation between *Gap* and *R*&*D*, indicating that there may be a nonlinear correlation between compensation gap and innovation. The correlation coefficients between the control variables were all less than 0.5, proving that there was no serious multicollinearity problem in the model.

### 4.2. Hierarchical Multiple Regression Results

**Figure 1:**
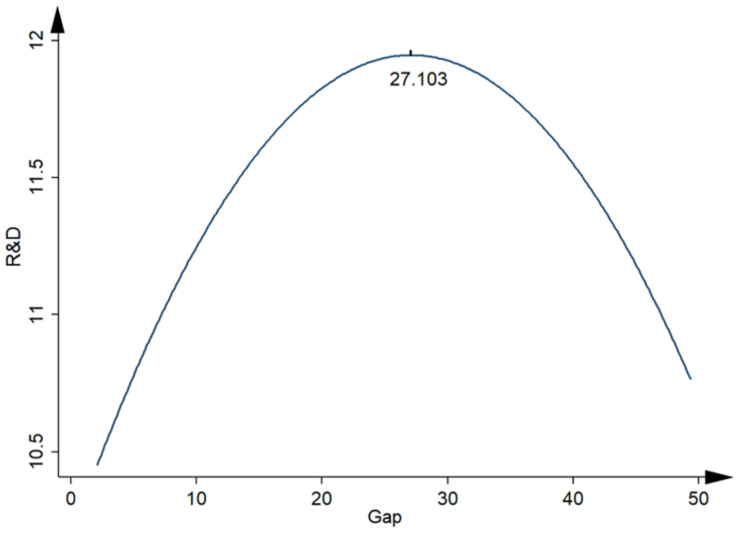
Inverted-U-shaped relationship between compensation gap and corporate innovation.

Table 3 shows the regression results of the impact of the internal compensation gap on the innovation of pharmaceutical enterprises. As displayed in Column 1, the regression coefficient of *Gap* was positively significant at the 10% level, which demonstrates that the internal compensation gap can promote *R*&*D* investment. Column 2 presents the result after *Gap*^2^ is added into the model, where the regression coefficient of *Gap* is positively significant at the level of 1%, whereas that of *Gap*^2^ is negatively significant at the same level. This preliminarily verifies the inverted-U-shaped relationship between the internal compensation gap and the innovation of pharmaceutical enterprises. To further verify this relationship, the UTEST test was conducted on the basis of the regression results. The extreme value was calculated to be 27.103, within the range of *Gap* (2.108, 49.32), and the result of the *t* test was 2.33, which again proves the existence of the inverted-U-shaped relationship, as shown in Figure 1. These regression results provide empirical evidence for Hypothesis 1, indicating that innovation investment in pharmaceutical enterprises tends to increase and then decrease as the compensation gap increases, and the stimulation effect reached the maximum when the compensation of executives was 27.103 times that of employees. In terms of control variables, companies that are younger, or companies with smaller size, lower institutional ownership, higher ROA, and lower asset turnover ratio may invest more in *R*&*D* investment.

### 4.3. Robustness Tests

The following tests were also conducted in order to further test the robustness of the conclusions. Measuring key variables in different methods is widely adopted to test for robustness. According to this, the measurement of the internal compensation gap was replaced with the natural logarithm of the difference between the average compensations of executives and employees. The above tests were repeated, obtaining the regression results as demonstrated in Table 4. In Column 1, the regression coefficient of *Gap* is positively significant at the level of 1%. After *Gap*^2^ had been added to the model, as shown in column (2), the regression coefficient of *Gap* was still positively significant at the level of 1%, whereas that of *Gap*^2^ was negatively significant at the same level. Significance remained consistent with the previous results, which further proves that there is an inverted-U-shaped relationship between compensation gap and innovation.

However, the following endogenous problem may exist in the above studies: the enterprise with strong willingness to innovate and an aggressive investment strategy is more likely to provide high compensation to attract executives. Hence, we solve this problem by the regression of the lagged dynamic panel model. In detail, all explaining variables were regressed again after lagging for one and two phases. Results are listed in Table 5. After lagging for one and two phases, the significance of *Gap* and *Gap*^2^, respectively, was still consistent with the above results. It is evident that the research conclusion is still robust after potential endogenous problems are eliminated.

### 4.4. Heterogeneity Test

#### 4.4.1. Heterogeneity of Marketization Level

The level of marketization reflects the comprehensive economic, social, and legal progress of different regions, and is an important reflection of the market efficiency of resource allocation [54]. Due to the different reform processes and levels of economic development in different provinces, the level of marketization varies widely across China’s provinces. Following the existing literature [55,56,57], the marketization level is measured by the marketization index, which was construed by Wang et al. [58], since they measured marketization from five aspects: commodity market, factor market, nonstate (private) sector, market intermediaries, and the relationship between the government and the market. All samples were divided into groups of high or low level of marketization. If the marketization index of the local region of the sample enterprise exceeded the national median level of the year, it implies that this region had a high marketization level; otherwise, the region had a low marketization level. The two groups of data were regressed, respectively, with results as illustrated in Table 6.

The inverted-U-shaped relationship between the compensation gap and innovation is more significant in enterprises that are situated in regions with a low level of marketization. The reason could be that the pharmaceutical industry is subject to government control and industry protection, and thus, its compensation system may be largely restricted by external forces. In slowly marketized regions, the government intervenes strongly in market economic activity, and thus, the internal information and operation mechanism of the enterprise can be easily observed by external investors and market regulators. Under a strong supervision, the compensation gap within the enterprise can be kept at a reasonable level, and the fairness and effectiveness of the remuneration contract mechanism is enhanced. As a result, in regions with a low level of marketization, the innovative incentive effect of the compensation system can be given full play. The corporate compensation system compensates, to a certain extent, for the lack of marketization level.

#### 4.4.2. Heterogeneity of Corporate Scale

To further test whether the results differed among enterprises of different scales, all sample enterprises were divided into a group of large enterprises and a group of small- and medium-sized enterprises according to whether their operating revenues exceeded the median operating revenues of the industry in that year; then, regressions were separately conducted. Results are presented in Table 7. The inverted-U-shaped relationship between the compensation gap and innovation of large enterprises was significant, whereas that of small/medium-sized enterprises was not. This is because, for pharmaceutical enterprises, *R*&*D* and innovation are long-term investment projects with high risk. Once *R*&*D* fails, the enterprise faces high costs. Compared with small- and medium-sized enterprises, large enterprises can take a high level of risk, and are more willing to innovate. In addition, large enterprises have more abundant innovative resources; therefore, as a result of compensation incentives, executives working in such enterprises are more likely to gain competitive advantages through rationally allocating such resources, and improving innovation abilities and market reputation. By contrast, small- and medium-sized enterprises are generally less willing to innovate for maintaining a long service life. Thereby, the stimulation effect of the compensation gap on innovation is more significant in large pharmaceutical enterprises.

## 5. Conclusions

Different from a general high-tech industry, the development of the pharmaceutical industry not only contributes to improving the level of scientific and technological innovation, but is also of great significance in satisfying people’s medical demands, and guaranteeing people’s living quality. As a typical technology- and capital-intensive industry, efficient *R*&*D* is the core driver to realize the pharmaceutical industry’s continuous competitiveness and growth. However, currently, research on how to effectively stimulate innovation in this industry is still in its infancy. From the combined effect of tournament theory and social comparison theory, this paper empirically verified the nonlinear relationship between internal compensation gap and corporate innovation, taking the listed pharmaceutical enterprises in China during 2011–2018 as the sample. Our results show that there is an inverted-U-shaped relationship between compensation gap and innovation; there is also a threshold between them, proving that the role of compensation gap is not endless. The best stimulation effect can be realized when the average compensation of executives is about 27 times that of employees; this relationship is more significant in enterprises located in less marketized regions, and large enterprises. These conclusions were still valid after being subjected to a series of robustness tests.

The following are recommendations for pharmaceutical enterprises and policy-makers. First, a rational enterprise can adjust its compensation system to stimulate innovation. A reasonable compensation scheme can be formulated in combination with the research conclusions and the industrial characteristics to maintain the enterprise’s compensation gap at such a suitable level that can improve employees’ working enthusiasm while stimulating the executive’s working ability to promote innovation. However, some enterprises are not clearly aware of this issue due to their restricted strategic vision and exogenous constraints. For such enterprises, the regulator can exogenously intervene to provide more innovation incentives.

Second, the compensation incentive mechanism should be adjusted as per the specific conditions of different enterprises. Our study indicates that the compensation gap cannot effectively stimulate innovation in pharmaceutical enterprises located in highly marketized regions, and small- and medium-sized pharmaceutical enterprises. For this reason, when enterprises are in a highly marketized region, the policy-maker should further enhance external supervision. Enterprises also need to improve the transparency of information disclosure, strengthen internal supervision, and restrict self-interested behavior of the manager, so as to guarantee the effective exertion of the compensation incentive policy. For small- and medium-sized enterprises, the policy-maker should provide a better platform for accessing innovation-related resources, and complete the intellectual property protection system to increase the enterprises’ level of risk-taking. In the meantime, enterprises should perfect the incentive mechanism by adopting a multi-incentive system (such as compensation, equity, and promotion incentives) and other means to enhance the willingness of managers and employees to innovate.

Nevertheless, this research has certain limitations. First, the research only focused on a single incentive method, namely monetary compensation under a compensation contract. As an increasing number of enterprises implement equity-incentive and stock-option plans, future research can focus on analyzing the existence of any differences in the effect of different stimulation methods on stimulating executives to innovate, and even analyzing whether company-paid consumption, job promotion, and other concealed incentives have a similar effect, as such analyses would be worthy and interesting. Moreover, different types of employees are responsible for different tasks in the enterprise, such as operation, production, management, and *R*&*D*. However, China’s listed pharmaceutical enterprises have not disclosed the compensation of different types of employees. We look forward to future studies that will provide a detailed classification of employees in order to explicitly and accurately explain the contributions of different employees to innovation, and put forward specific incentive schemes to efficiently stimulate innovation.

## Figures and Tables

**Table 1:** Variable measurement.

Variables	Measurement	Expected Sign
Panel A: Dependent variables		
*R*&*D*	Ratio of *R*&*D* investment to total revenues	
Panel B: Independent variables		
*Gap*	Ratio of average executive compensation to average employee compensation	+
Panel C: Control variables		
*Insto*	Number of shares held by institutional investors divided by the total shares in issue	−
*Age*	Difference between year (t) and the year when firm (i) was established	−
*Assets*	Natural logarithm of book value of total assets plus one	+
*ROA*	Return on assets, which equals to net income divided by total assets	+
*Lev*	Book value of total debts divided by the book value of total assets	−
*Cash*	Book value of cash holdings divided by the book value of total assets	+
*Turn*	Book value of total revenues divided by the book value of total assets	−
*Sales*	Increased percentage of sales	−
*Year*	Dummy variables of years	
*Province*	Dummy variables of provinces	

**Table 2:** Descriptive statistics and correlations.

Variables	N		Mean		SD		Min		Max	
Panel A: Descriptive statistics										
*R*&*D*	1520		4.281		2.874		0.179		15.90	
*Gap*	1520		10.49		7.622		2.108		49.32	
*Insto*	1520		42.48		22.13		0.274		90.05	
*Age*	1520		18.09		5.559		4		37	
*Assets*	1520		22.00		0.902		20.07		24.14	
*ROA*	1520		7.228		6.08		−11.79		23.43	
*Lev*	1520		31.59		17.44		4.087		72.15	
*Cash*	1520		2.067		3.476		0.105		23.78	
*Turn*	1520		64.06		58.12		12.74		560.7	
*Sales*	1520		15.31		21.9		−37.43		105.4	
Panel B: Correlations										
	1	2	3	4	5	6	7	8	9	10
1. *R*&*D*	1									
2. *Gap*	0.0170	1								
3. *Insto*	−0.093 **	0.189 ***	1							
4. *Age*	−0.0260	0.0580	0.289 ***	1						
5. *Assets*	0.062 *	0.387 ***	0.387 ***	0.283 ***	1					
6. *ROA*	0.0560	0.190 ***	0.247 ***	0.0330	0.0550	1				
7. *Lev*	−0.125 ***	−0.0160	0.0590	0.071 *	0.230 ***	−0.436 ***	1			
8. *Cash*	0.0510	−0.0300	−0.0130	−0.159 ***	−0.153 ***	0.197 ***	−0.53 ***	1		
9. *Turn*	−0.263 ***	0.0230	0.0250	−0.0340	−0.063 *	0.127 ***	0.055	−0.101 ***	1	
10. *Sales*	0.0400	0.093 **	0.00900	−0.100 ***	0.00600	0.223 ***	−0.020	−0.076 **	0.005	1

Note: ***, **, and * represent significance at the 1%, 5%, and 10% levels, respectively.

**Table 3:** Impact of internal compensation gap on innovation input.

	(1)	(2)
Variables	*R*&*D*	*R*&*D*
*Gap*	0.027 *	0.130 ***
	(1.95)	(3.48)
*Gap* ^2^		−0.002 ***
		(−3.01)
*Insto*	−0.016 ***	−0.017 ***
	(−3.01)	(−3.20)
*Age*	−0.040 **	−0.041 **
	(−2.11)	(−2.17)
*Assets*	−0.273 *	−0.318 **
	(−1.93)	(−2.27)
*ROA*	0.068 ***	0.066 ***
	(3.07)	(3.04)
*Lev*	−0.003	−0.001
	(−0.47)	(−0.17)
*Cash*	0.013	0.013
	(0.40)	(0.39)
*Turn*	−0.014 ***	−0.014 ***
	(−9.59)	(−9.86)
*Sales*	0.000	−0.000
	(0.04)	(−0.05)
*Year fixed effect*	YES	YES
*Province fixed effect*	YES	YES
*Constant*	9.820 ***	10.189 ***
	(3.31)	(3.47)
Observations	1520	1520
Adjusted R^2^	0.273	0.280

Note: ***, **, and * represent significance at the 1%, 5%, and 10% levels, respectively.

**Table 4:** Robustness test: different measures of internal compensation gap.

	(1)	(2)
Variables	*R*&*D*	*R*&*D*
*Gap*	0.724 ***	9.792 ***
	(4.90)	(3.67)
*Gap* ^2^		−0.339 ***
		(−3.39)
*Insto*	−0.018 ***	−0.018 ***
	(−4.01)	(−4.07)
*Age*	−0.051 ***	−0.050 ***
	(−2.73)	(−2.72)
*Assets*	−0.059	−0.019
	(−0.45)	(−0.14)
*ROA*	0.040	0.040
	(1.60)	(1.61)
*Lev*	−0.006	−0.005
	(−0.94)	(−0.75)
*Cash*	0.010	0.015
	(0.33)	(0.50)
*Turn*	−0.013 ***	−0.014 ***
	(−9.68)	(−9.71)
*Sales*	0.003	0.004
	(0.63)	(0.80)
*Year fixed effect*	YES	YES
*Province fixed effect*	YES	YES
*Constant*	−2.611	−63.961 ***
	(−1.01)	(−3.55)
Observations	1520	1520
Adjusted R^2^	0.143	0.150

Note: *** represents significance at the 1% level.

**Table 5:** Robustness test: one- and two-year lag of explanatory variables.

	(1)	(2)	(3)	(4)
Variables	*R*&*D*	*R*&*D*	*R*&*D*	*R*&*D*
*Gap_i,t−1/t−2_*	0.027 *	0.132 ***	0.027 *	0.170 ***
	(1.80)	(3.19)	(1.81)	(3.79)
*Gap_i,t−1/t−2_^2^*		−0.002 ***		−0.003 ***
		(−2.91)		(−3.97)
*Insto_i,t−1/t−2_*	−0.015 **	−0.016 ***	−0.017 ***	−0.018 ***
	(−2.49)	(−2.66)	(−2.67)	(−2.89)
*Age_i,t−1/t−2_*	−0.033 *	−0.035 *	−0.035	−0.037 *
	(−1.68)	(−1.73)	(−1.64)	(−1.68)
*Assets_i,t−1/t−2_*	−0.273 *	−0.309 **	−0.289 *	−0.328 **
	(−1.77)	(−2.02)	(−1.73)	(−2.00)
*ROA_i,t−1/t−2_*	0.087 ***	0.085 ***	0.110 ***	0.106 ***
	(3.62)	(3.55)	(4.12)	(3.96)
*Lev_i,t−1/t−2_*	−0.002	−0.000	0.003	0.005
	(−0.30)	(−0.06)	(0.42)	(0.60)
*Cash_i,t−1/t−2_*	0.029	0.028	0.045	0.043
	(0.88)	(0.82)	(1.31)	(1.21)
*Turn_i,t−1/t−2_*	−0.014 ***	−0.014 ***	−0.015 ***	−0.015 ***
	(−9.40)	(−9.77)	(−8.83)	(−9.40)
*Sales_i,t−1/t−2_*	0.004	0.003	0.002	0.002
	(0.78)	(0.68)	(0.42)	(0.30)
*Year fixed effect*	YES	YES	YES	YES
*Province fixed effect*	YES	YES	YES	YES
*Constant*	9.732 ***	9.921 ***	10.115 ***	10.183 ***
	(3.02)	(3.10)	(2.87)	(2.93)
Observations	1330	1330	1140	1140
Adjusted R^2^	0.267	0.273	0.268	0.281

Note: ***, **, and * represent significance at the 1%, 5%, and 10% levels, respectively.

**Table 6:** Heterogeneity test: high level of marketization vs. low level of marketization.

	High Level of Marketization		Low Level of Marketization	
	(1)	(2)	(3)	(4)
Variables	*Input*	*Input*	*Input*	*Input*
*Gap*	0.016	0.048	0.082 ***	0.412 ***
	(0.91)	(1.09)	(3.06)	(6.70)
*Gap* ^2^		−0.001		−0.007 ***
		(−0.77)		(−6.28)
*Insto*	−0.012 *	−0.012 *	−0.019 **	−0.031 ***
	(−1.82)	(−1.81)	(−2.23)	(−3.72)
*Age*	−0.017	−0.018	−0.027	−0.006
	(−0.78)	(−0.84)	(−0.35)	(−0.08)
*Assets*	−0.033	−0.056	−1.487 ***	−1.476 ***
	(−0.21)	(−0.35)	(−3.75)	(−4.24)
*ROA*	0.088 ***	0.090 ***	0.061	0.008
	(3.87)	(3.92)	(1.23)	(0.17)
*Lev*	−0.006	−0.005	0.022	0.013
	(−0.80)	(−0.63)	(1.57)	(1.11)
*Cash*	−0.003	−0.002	0.068	0.049
	(−0.07)	(−0.05)	(1.50)	(0.97)
*Turn*	−0.013 ***	−0.013 ***	−0.045 ***	−0.045 ***
	(−10.53)	(−10.59)	(−4.49)	(−4.80)
*Sales*	−0.002	−0.003	0.004	0.005
	(−0.46)	(−0.51)	(0.59)	(0.74)
*Year fixed effect*	YES	YES	YES	YES
*Province fixed effect*	YES	YES	YES	YES
*Constant*	4.051	4.322	42.264 ***	40.656 ***
	(1.20)	(1.29)	(4.98)	(5.34)
Observations	1152	1152	368	368
Adjusted R^2^	0.246	0.246	0.443	0.531

Notes: ***, **, and * represent significance at the 1%, 5%, and 10% levels, respectively.

**Table 7:** Heterogeneity test: large-scale enterprises vs. small- and medium-scale enterprises.

	Large-Scale Enterprises		Small- and Medium-Scale Enterprises	
	(1)	(2)	(3)	(4)
Variables	*Input*	*Input*	*Input*	*Input*
*Gap*	0.027 *	0.125 ***	−0.039	0.100
	(1.78)	(2.97)	(−0.48)	(0.49)
*Gap* ^2^		−0.002 **		−0.007
		(−2.54)		(−0.85)
*Insto*	−0.018 ***	−0.019 ***	0.014	0.013
	(−3.17)	(−3.34)	(0.95)	(0.90)
*Age*	−0.021	−0.022	0.705 *	0.713 *
	(−0.95)	(−0.99)	(1.96)	(1.96)
*Assets*	−0.354 *	−0.395 **	−0.092	−0.093
	(−1.80)	(−2.04)	(−0.10)	(−0.10)
*ROA*	0.085 ***	0.085 ***	−0.031	−0.032
	(3.33)	(3.34)	(−0.81)	(−0.82)
*Lev*	0.005	0.005	0.020	0.022
	(0.56)	(0.68)	(1.39)	(1.43)
*Cash*	−0.017	−0.017	0.006	0.009
	(−0.55)	(−0.53)	(0.08)	(0.12)
*Turn*	−0.014 ***	−0.015 ***	−0.009	−0.010
	(−9.64)	(−9.85)	(−0.83)	(−0.91)
*Sales*	−0.004	−0.004	−0.011 *	−0.011 *
	(−0.85)	(−0.88)	(−1.72)	(−1.67)
*Year fixed effect*	YES	YES	YES	YES
*Province fixed effect*	YES	YES	YES	YES
*Constant*	11.093 ***	11.388 ***	−5.024	−5.725
	(2.71)	(2.82)	(−0.21)	(−0.23)
Observations	1328	1328	192	192
Adjusted R^2^	0.269	0.274	0.732	0.730

Note: ***, **, and * represent significance at the 1%, 5%, and 10% levels, respectively.

## Data Availability

China Stock Market and Accounting Research Database (CSMAR): https://www.gtarsc.com/ (accessed on 1 December 2020).
